# Epigraph: A Vaccine Design Tool Applied to an HIV Therapeutic Vaccine and a Pan-Filovirus Vaccine

**DOI:** 10.1038/srep33987

**Published:** 2016-10-05

**Authors:** James Theiler, Hyejin Yoon, Karina Yusim, Louis J. Picker, Klaus Fruh, Bette Korber

**Affiliations:** 1 Los Alamos National Laboratory, Los Alamos, NM 87545, USA; 2 New Mexico Consortium, Los Alamos, NM 87544, USA; 3 Oregon Health and Science University, Portland, OR 97239, USA

## Abstract

Epigraph is an efficient graph-based algorithm for designing vaccine antigens to optimize potential T-cell epitope (PTE) coverage. Epigraph vaccine antigens are functionally similar to Mosaic vaccines, which have demonstrated effectiveness in preliminary HIV non-human primate studies. In contrast to the Mosaic algorithm, Epigraph is substantially faster, and in restricted cases, provides a mathematically optimal solution. Epigraph furthermore has new features that enable enhanced vaccine design flexibility. These features include the ability to exclude rare epitopes from a design, to optimize population coverage based on inexact epitope matches, and to apply the code to both aligned and unaligned input sequences. Epigraph was developed to provide practical design solutions for two outstanding vaccine problems. The first of these is a personalized approach to a *therapeutic* T-cell HIV vaccine that would provide antigens with an excellent match to an individual’s infecting strain, intended to contain or clear a chronic infection. The second is a pan-filovirus vaccine, with the potential to protect against all known viruses in the *Filoviradae* family, including ebolaviruses. A web-based interface to run the Epigraph tool suite is available (http://www.hiv.lanl.gov/content/sequence/EPIGRAPH/epigraph.html).

## Introduction

HIV is highly variable, largely as a consequence of immune selection acting on this highly mutable virus during chronic infection^1^,^2^,^3^,^4^; even the most conserved regions of HIV are variable at the epitope level^5^,^6^. T-cell epitopes are short contiguous stretches of protein, peptides generally between 9–12 amino acids long, which are presented on the surface of infected cells to enable recognition, and to trigger a T-cell based immune-response. Epitope variability limits the cross-reactive potential of single antigen vaccines, such as a natural protein or a consensus sequence^7^,^8^. Mosaic vaccines were originally designed to contend with HIV diversity by including a small set of (typically two to four) complementary antigens, rather than a single antigen. They include several artificial proteins that resemble natural proteins, but are collectively designed to maximally cover diverse epitopes in a targeted population^9^, offering highly improved epitope coverage over combinations of natural sequences.

Mosaics and Epigraphs solve essentially the same optimization problem (PTE coverage), and are thus expected to behave the same way experimentally. Mosaic antigens have already been designed, synthesized, and tested, and have shown promise on a variety of fronts. When expressed, Mosaic antigens have folded well in terms of binding discontinuous antibodies, and are highly immunogenic, eliciting both T-cell and antibody responses^8^,^10^,^11^. T-cell responses induced by Mosaics effectively target HIV infected cells^12^, and are more cross-reactive than those induced by natural proteins^8^,^10^,^13^,^14^,^15^. Mosaic vaccines have shown promise against HIV-1^8^,^10^,^11^,^13^,^16^, as well as other variable pathogens, including the viruses that cause Hepatitis C^17^, Ebola^18^, and Influenza^19^.

Despite the similarities in the overall optimization criteria, Epigraphs provide substantial advantages over our original Mosaic strategy. Mosaics use a genetic algorithm^9^, while Epigraphs use a much faster graph-based approach (see Formulation). This speed, as well as the structure of the mathematical framework, facilitated the addition of new features to the Epigraph tool suite^20^. More importantly, while Mosaics provided a near optimal solution for antigen design to maximize PTE coverage by a vaccine across a simple population, the code was not readily adapted to more complex problems. We developed Epigraph to enable computational solutions to two pressing T cell vaccine design problems that were intractable using the computationally slower Mosaic algorithm: a pan-filovirus T-cell vaccine and a strategy for matching vaccines to infecting strains in a therapeutic setting.

Recently there has been resurgence of interest in T cell-based vaccines. SIV vaccine antigens presented in rhesus macaque cytomegalovirus (RhCMV) vectors generate prolific T-cell responses that enable stringent control and progressive clearance of pathogenic SIV upon infection in over 50% of vaccinated monkeys. These responses violate traditional paradigms of T cell-mediated immunity, and provide new impetus for exploring T-cell vaccine approaches^21^,^22^. There is also keen interest in focusing vaccine-stimulated T-cell responses on conserved regions, to shift immunodominance to epitopes with a limited capacity to escape because they are under fitness constraints^16^,^23^,^24^,^25^. Such T-cell vaccination strategies may be beneficial in either a preventive or therapeutic setting. It was to pursue these innovative vaccine strategies that we developed Epigraph, a flexible and computationally efficient strategy for optimizing epitope coverage in a variety of scenarios. The coupling of Epigraph antigen design with contemporary vaccine delivery approaches offers a promising strategy with the potential to advance vaccine efforts against the challenge of highly variable pathogens.

Here we applied Epigraph to provide potential solutions to two outstanding vaccine design problems. First, we describe a Tailored Therapeutic Vaccine (TTV) approach. In contrast to a vaccine that prevents infection, in therapeutic setting it is possible to obtain sequences from the infected person who will be treated, and attempt to match their infecting strain as closely as possible to the vaccine. It is not currently feasible to make a new matched vaccine for every person you treat. The TTV approach enables the design of a small set (a half a dozen or so) vaccine antigens, a practical number for manufacture. Then, for each individual, the best two or three antigen subset of these six can be selected to provide a “tailored” match to viral sequences sampled from the patient to be treated. The Epigraph-based TTV code optimizes the set of vaccine antigens for manufacture, such that the set will sample the diversity of the target population, and enable the best vaccine matches overall for infected individuals in the target population. Here we apply TTV approach to HIV, though the strategy is general. A single TTV design run can loop over more than a thousand basic Epigraph runs, so the computational efficiency is essential to complete the Epigraph design.

We next explored the problem of how to design a vaccine that could cover the viral diversity found in the entire *Filoviradae* family, which includes ebolavirus and marburgvirus viruses, as well as other related viruses that can cause fatal hemorrhagic fevers in human and non-human primates. First we used Epigraph to define the most conserved regions of the filovirus proteome, then we used it to design antigens that would best cover the diversity that was found in those regions. We explored dozens of design strategies to finally identify a vaccine solution that met three criteria. First, we felt it was critical that the potential T-cell epitope coverage of the ebola virus species, that has historically seeded most outbreaks, not be compromised. Second, we wanted the design to have the potential to elicit responses against of the full range of known diversity of viruses in the *Filoviradae* family. Third, we made sure that the conserved regions that were included spanned relatively large stretches of protein, so that epitopes representing a broad spectrum of human leukocyte antigens (HLAs) would be included. Exploring the combinatorics of the many design options we considered to meet these criteria would have been prohibitive using the slower Mosaic code, but through systematic use of Epigraph, we were able to identify a promising design strategy that met our 3 criteria.

### Epigraph Formulation

Central to both Epigraphs and Mosaics is the concept of potential epitope coverage. Because *known* T-cell epitopes are very densely packed in HIV^5^, we consider every contiguous epitope-length fragment (*i.e.,* every *k*-mer) to be a *potential* epitope. We usually set *k* = 9 as the length of potential T-cell epitopes (PTEs), as this is the optimal length of most cytotoxic T-cell Class I presented epitopes^5^,^9^, but solutions optimized on *k* = 9 are still very good for other common epitope lengths of 8–12 amino acids (Supplementary Table 1). If using a PTE length of 9, the first PTE in a sequence will be the peptide from position 1 to 9 in the protein, the second PTE from 2 to 10, *etc.*

**Figure 1:**
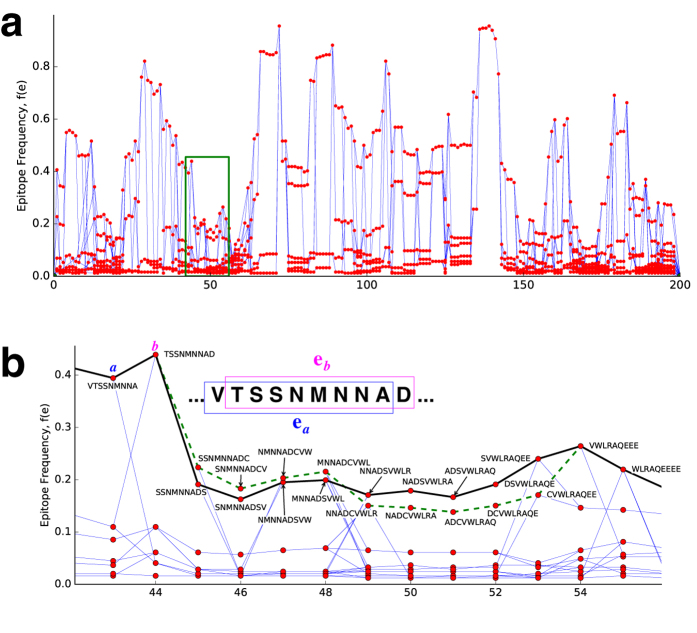
**(a)**Full graph for the CRF01-AE clade of the Nef protein. The green rectangle is an inset shown in (B). Nodes are red dots, and represent each *k*-mer variant, with *k* = 9. The edges are thin blue lines that connect epitopes whose sequences overlap by *k* − 1 amino acids, as shown for the first two epitopes (**e**_*a*_ = VTSSNMNNA, **e**_*b*_ = TSSNMNNAD) in the upper left of (B). Although the topological properties of the graph do not depend on the node positions, this plot uses the vertical axis to indicate epitope frequency in the target sequence set, *y* = *f*(**e**), for each node. The horizontal position of the nodes is chosen so that all directed edges connect from left to right. The ideal path through this graph keeps as much as possible to the largest *y*-values; this path defines a protein sequence that maximizes epitope coverage of the population. **(b)** The inset shows two paths through the nodes. The solid black line is the optimal path, and corresponds to the sequence VTSSNMNNAD**S**VWLRAQEEEE while the dashed green corresponds to VTSSNMNNAD**C**VWLRAQEEEE. The dashed line achieves higher *f*(**e**) values on 4 nodes, but the solid line has higher *f*(**e**) for 5 nodes, and ∑ *f*(**e**) is higher. Note there is no path that includes the highest-valued nodes for all horizontal positions.

Here we will briefly describe the steps taken in the Epigraph algorithm, to impart an intuition for what the algorithm is doing; a more detailed and precise mathematical description is provided in the Methods. Figure 1 provides an illustration of the Epigraph strategy.

The first step in Epigraph design is to assemble a representative sample of *N* protein sequences that embodies the viral diversity in a population that will be targeted for vaccine use (*e.g.,* a phylogenetic clade, a country, or the world). The input proteins do not have to be aligned, but they can be, as will be discussed below. Each sequence in the set is decomposed into all possible 9-mers, and the number, *n*, of recurrences of a particular 9-mer in the sample population is tallied. Each unique 9-mer found at least once in the sample population will be associated with its frequency in the population. For instance, if there are 1000 sequences in the sample population (*N* = 1000), and a particular 9-mer was found exactly matched in 200 of those sequences (*n* = 200), then the frequency of that 9-mer is *n*/*N* = 0.2. We characterize the potential cross-reactivity of an antigen by the sum of the frequencies of all the 9-mers in the antigen sequence; if we divide this quantity by the sum of frequencies of all the distinct 9-mers in the population, that provides the coverage score.

Next, as illustrated in Fig. 1, a graph is created. Formally, a graph is a collection of nodes and edges (edges connect pairs of nodes). In our graph, each node corresponds to a unique 9-mer, and two nodes are connected by an edge whenever the 9-mers in those two nodes share an overlap of 8 amino acids. A path through this graph is a sequential assembly of connected nodes, with the last 8 amino acids of each node matching the first 8 amino acids of the subsequent node. These overlaps allow such a path to be associated with a single sequence of amino acids. Epigraph (implicitly) considers all the paths – there are exponentially many of them – in the graph and identifies an optimal path. The criterion for optimality is the coverage score, which is proportional to the sum of frequencies associated with the nodes in the path. And from these nodes, we can construct an intact full-length protein sequence. This sequence is the first antigen in our vaccine, and it will contain the most common 9-mers in the target population, to the extent possible given the constraint that those 9-mers have to overlap so that they can be expressed with a single complete protein sequence. For a monovalent vaccine, this antigen is all we need. But once the first antigen has been generated, we can produce a second complementary sequence by finding a second path through the graph that again optimizes the sum of frequencies, but this time without including the frequencies of 9-mers that were already included in the first antigen. This second step is achieved by setting the frequencies of those initial 9-mers to zero during this second optimization. In this way, if a particular 9-mer is an essential block for building a complete protein, it can be incorporated into both antigens, but as it does not increase the coverage score, it will not be favored. This process is repeated until the desired number of antigens is generated for a polyvalent vaccine.

## Comparison of Epigraph with Mosaics

The Mosaic approach optimizes coverage with a genetic algorithm in a loop that alternately recombines regions of natural proteins at random breakpoints and creates pools of these *in silico* generated recombinants, and then selects those candidates with the best coverage for the next generation from among those pools^9^. In contrast, the Epigraph algorithm optimizes that criterion by finding a path through the *k*-mer overlap graph (Fig. 1). Epigraph solutions generally have a slightly improved PTE population coverage relative to Mosaics when applied to HIV proteins (Supplementary Tables 2 and 3). While this coverage advantage is small, the computational advantage in terms of run-time is substantial. Epigraph can complete a basic vaccine design in seconds on a laptop (Supplementary Table 8), while Mosaic designs can take hours to days to approach optimization^26^. When Mosaic antigens are used to initialize an Epigraph run, coverage scores can often be increased, albeit very slightly (Supplementary Table 4). Thus high quality (and in certain cases, mathematically optimal) antigens can be very rapidly determined with Epigraph, and this leads to new opportunities for innovative vaccine design that would otherwise be computationally onerous to pursue.

Two caveats are in order here. One is that an Epigraph solution is mathematically optimal only if the directed graph is acyclic–that is to say, the graph generated in Fig. 1 contains no cycles. One simple example of how a cycle can arise in a graph is when a 9-mer is precisely repeated in two different places in a protein. In practice, most graphs we have used are not acyclic, and that usually means we need to do some pre-processing (see Methods: De-cycling). A second caveat is that optimality only applies to the single antigen (monovalent) case. For polyvalent vaccines, we employ heuristics to bootstrap the monovalent optimality (see Methods: Polyvalent vaccines).

## Excluding Rare Epitopes

Natural but rare PTEs are undesirable in a vaccine because they can elicit type-specific responses. Natural HIV proteins carry a surprising number of such PTEs, and when these rare forms are immunogenic or immunodominant, they may curtail the cross-reactive potential of a vaccine. One of the analysis tools in the Epigraph tool suite^20^ provides the frequencies of every distinct PTE in a population, and the output provides a sobering lens with which to view natural HIV diversity. For example, the Los Alamos HIV database M group Env alignment^27^ of 4,250 sequences contains over 650,000 distinct 9-mer peptides; of these, over 500,000 are unique, each appearing only once in the population. This is an average of 120 unique PTEs per natural Env sequence, and responses to such PTEs would likely be strain-specific. Even among Gag sequences, one of the most conserved HIV proteins, there are just under 129,000 distinct naturally found 9-mers found in 4,596 M group sequences, over 60% of which appear only once, an average 18 completely unique 9-mers per natural sequence.

**Figure 2:**
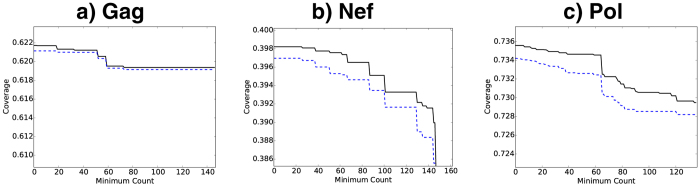
Excluding rare epitopes. We see that excluding rare variants decreases the coverage, but only slightly. Coverage of polyvalent (*m* = 2) solutions is shown as a function of minimum count *n*_*o*_. These graphs are created by sequentially increasing *n*_*o*_ and eliminating all nodes **e** from the graph for which *f*(**e**) ≤ *f*_*o*_ = *n*_*o*_/*N*, where *N* is the number of sequences in the sample population set. This continues until the maximum *n*_*o*_ is achieved for which a path still exists from Begin to End. Note that this maximum value can be computed directly from the graph, before this sequential process is employed. Blue dashed lines correspond to coverage given by the direct sequential algorithm; the black solid lines are based on the best solutions after 100 random restarts. To facilitate comparison, the vertical axis, in all three plots, is restricted to a range of 0.015.

While Epigraphs generally disfavor rare epitopes, we can constrain solutions to strictly avoid them. The Epigraph tool suite^20^ directly enables users to investigate the relationship between PTE coverage and rare epitope exclusion. To illustrate this, Epigraph solutions based on global HIV database protein alignments were obtained for HIV proteins Env, Gag, Pol and Nef (Fig. 2). Because Env includes hypervariable regions, inclusion of some rare epitopes is required for Epigraph antigens to create a complete protein: its largest cutoff is *n*_*o*_ = 2, so for a complete Env to be generated, some PTEs must be included that are only found three times in the full database. By contrast, Gag, Nef, and Pol antigens can readily be constructed for *n*_*o*_ > 100 (that is, the rarest epitope in the vaccine appears in over 100 target population sequences). As Fig. 2 illustrates, this can be accomplished with minimal PTE coverage loss, and thus merits consideration in future vaccine designs. In practice, the vaccine designer can create this graph of coverage versus *n*_*o*_ and based on this trade-off, select a threshold *n*_*o*_ to use for a final Epigraph run.

For practical use, particularly with a large number of input sequences in the target population, we recommend using a cutoff of at least *n*_*o*_ = 1, so that each PTE included in the Epigraph sequences appears more than just once in the sample target population. But users are encouraged to use a larger cutoff, as long as there is negligible cost in terms of coverage. Excluding rare variants also speeds up the computation time (Supplemental Table 8). We remark that for sample target populations with only a few target sequences – such as the Ebola set with only 32 distinct protein sequences – we required that epitopes only be present once in the set to be considered for inclusion in the Epigraph antigens, to improve coverage of all variants.

## Aligned and Unaligned Input Sequences

The default variant of the Epigraph algorithm does not require that the input sequences in the sample target population be aligned. The *k*-mer overlap graph depends only on the PTEs that are in the sequences, not on their positions in those sequences.

A variant of Epigraph was developed, however, that uses *aligned* target population sequences for input, and produces antigens on output that are aligned with these input sequences. The extra structure that is imposed on the aligned solution often, though not always, leads to slightly lower PTE coverage scores (see Supplementary Table 9). But an important consequence of this structure is that the aligned variant produces a graph that is, by construction, acyclic; this eliminates the need for a heuristic de-cycling step.

## Optimizing on Inexact Matches

A further advantage of the extra structure imposed by alignment is that it permits other variants of the antigen design algorithms that would be impractical with the open unaligned graph. One such extension is the optimization of coverage by inexact matches. The motivation here is that an antigen epitope may still be cross-reactive with a target epitope, even though they don’t exactly match. Instead of maximizing a coverage that gives credit to an antigen PTE only if it exactly matches a corresponding PTE in the sample target population, we modify our criterion to give credit for approximately matching PTEs in the target population. For example, if the antigen includes the 9-mer VTSSNMNNA, then it gets credit not only for every appearance of VTSSNMNNA in the target population, but also for appearances of VTSSNMNNC, VTSSNMNDA, *etc.*, which agree with the antigen 9-mer VTSSNMNNA in 8/9 of the positions. As we describe in the Supplement, we can optimize on inexact coverage and still give “bonus” credit to exact matches. We employ inexact-match coverage in our design of an Ebola vaccine, described below.

## Results

### Tailored Therapeutic Vaccines

A tailored therapeutic vaccine (TTV) is intended for a treatment situation in which the patient is already infected (hence, *therapeutic* instead of preventative), and a sample of a patient’s infecting viral quasispecies sequence is available (allowing the vaccine to be *tailored* to the patient’s specific infection). Given current technology and costs, it is not feasible to create a *de novo* vectored vaccine for every subject. It is feasible, however, to sequence a sample of each patient’s virus. Thus the more modest goal addressed here is to manufacture an affordable pool of *m* distinct vaccine antigens, from which only a subset (*n* < *m*) is delivered to each patient, with the subset chosen to maximize PTE coverage of the patient’s viral sequences by the selected Epigraphs.

Given the pool of *m* sequences, it is straightforward to select the best subset of *n* for each patient. Since *n* and *m* are small, one can quickly consider all possible subsets, and choose the one with maximum coverage of that patient’s sequences. The challenge is to construct the pool of *m* artificial antigens so that these *n*-out-of-*m* subsets are optimally effective.

A plausible but suboptimal approach is to create an *m*-valent Epigraph vaccine to optimize global coverage. A problem with this approach, especially for sequentially derived antigens, is that with each additional sequence, increasingly rare *k*-mers are included in later sequences, so the first *n* Epigraphs are typically the best choice for most individuals, offering no increase in coverage using a tailored approach over a simple Epigraph *n*-valent approach.

We explored several alternative strategies to achieve better coverage of sequences from the population of interest. The best of these, which is detailed in Algorithm 4, employs a clustering strategy. We start with a single antigen sequence **q**_*o*_ that Epigraph produces by optimizing coverage over the whole sample population set. This will be used as the first sequence in the manufactured set. We then partition the sample population sequences into *m* − 1 clusters, with the grouping based on PTE similarity scores (excluding the PTEs that were found in the initial sequence **q**_*o*_). Epigraph is separately applied to each cluster to obtain a centroid sequence for that cluster; *i.e.,* **q**_*i*_ is obtained by maximizing the coverage over the sequences in *i*'th cluster provided by the PTEs in the antigen set {**q**_*o*_, **q**_*i*_}. The process is iterative: the population sequence set is re-clustered by re-assigning each sequence to the **q**_*i*_ that maximally covers the PTEs in that sequence, and the new assignments lead to updated **q**_*i*_’s, and so on until convergence. Finally, {**q**_*o*_, **q**_1_, …, **q**_*m*−1_} is the set of *m* antigens that are manufactured.

The outcome can be sensitive to the initial conditions (clustering begins with initial random centroids), so we perform 100 complete runs using different starting sequences, and retain the best one as our solution. Given the number of iteration cycles in a single run, and the number of repeat runs with different initial conditions, the computational speed of the Epigraph algorithm is critical.

We applied TTV to three potential HIV target sequence populations for treatment studies: 189 contemporary B-clade sequences sampled in the United States, 199 contemporary C clade sequences sampled in Southern Africa, and 2015 Los Alamos HIV database 4596 global M-group Gag sequences (Supplementary Table 5). Gag was used for this pilot study because it is richly populated with beneficial epitopes in natural HIV infection^28^, and because SIV Gag responses are well-characterized in the context of RhCMV vector delivery^21^,^22^, and so it is a natural choice natural choice for inclusion in a CMV Tailored HIV vaccine. We evaluated p24 separately, because it is the most conserved region within Gag^5^.

**Figure 3:**
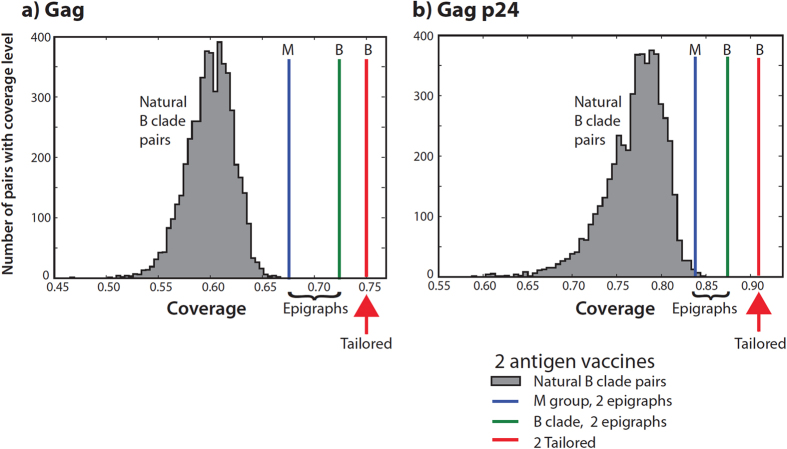
Two-antigen vaccine coverage. Comparisons illustrating the average epitope coverage per sequence of 189 B clade sequences isolated in the United States within the last decade, considered as a hypothetical target population for a tailored therapeutic vaccine (TTV). To illustrate PTE coverage using a pair of natural within-B clade sequences as vaccine antigens, 5000 randomly selected pairs of natural B clade sequences (gray) were evaluated as potential vaccines, and the distribution of average coverage of the sequences by natural pairs of antigens is shown in the gray histogram. This is compared to the average coverage provided by a two-antigen set of M group Epigraphs (M database, blue), a two-antigen set of global B clade Epigraphs (B database, green), and a US B clade TTV where the *n* = 2 best matches from a set of *m* = 6 representative Epigraphs for manufacture were chosen as a “tailored” match for each of the 189 natural B clade US sequences. The TTV antigens provide the best matches. Of note, the global M group two-antigen Epigraph solutions perform better than two natural B clade Gag proteins even in a within-clade setting, and the M group Epigraphs have the potential for a global response at or near this level of PTE coverage across all clades. **(A)** The comparisons for the full Gag protein, **(B)** The comparisons for only the conserved p24 region.

Figure 3 illustrates PTE coverage of contemporary B clade US Gag and p24 sequences by bivalent vaccines. B clade Epigraphs are markedly better than any combination of 2 natural B clade strains, while the best coverage was achieved by 2 TTV antigens selected from a pool of 6 (Fig. 3).

Extra epitopes in a vaccine that are not matched in the individual’s infection may trigger irrelevant responses, potentially diminishing vaccine-induced beneficial responses^16^. So we also monitored the extra PTEs in the TTVs. For given *n*, increasing the size of the manufactured pool from *m* = 2 to *m* = 6 increases coverage (by over ten percent for the M-group) without increasing the number of extra PTEs. Compared to the full-length Gag, the conserved-region p24 achieves improved coverage and dramatically reduced extras. (Fig. 3; also see Supplementary Table 5) However, Gag is ~500 amino acids in length, while p24 has only ~230 amino acids, so the increased coverage of p24 comes at the cost of encompassing fewer PTEs. Increasing the number vaccine antigens increases the epitope coverage with diminishing returns, and at the cost of including many more mismatched epitopes. For example, in Gag, increasing the number of tailored antigens from 1 to 2 to 3 increases PTE coverage from 61% to 75% to 79%, but also increases the average number of extra epitopes (in the vaccine but not in the target protein) from 196 to 537 to 773 (Supplementary Table 5).

### Filovirus/Ebola

Here we propose an Epigraph solution to a conserved-region pan-filovirus vaccine. The Epigraph tool was used for two purposes: first to define conserved regions within the proteome, and then to design the best combination of antigens within those regions for maximizing PTE coverage. A T-cell based Epigraph design may be particularly useful for viruses in the family *Filoviridae*, because vaccine-elicited T-cell responses to ebolaviruses are protective in non-human primates (NHPs)^29^,^30^, and filoviruses are highly diverse^31^,^32^,^33^. We assembled, annotated and aligned all available filovirus proteomes as we worked the vaccine project, and made the alignments available as part of our new HFV database^31^,^32^,^33^.

Viruses in the *Filoviridae* family have caused nearly 50 outbreaks in humans since their discovery in 1967, the most recent of which was the devastating 2014 West African epidemic^34^,^35^. There are five distinct species in the *Ebolavirus* genus virus: Ebola virus (EBOV), Sudan virus (SUDV), Reston virus (RESTV), Taï Forest virus (TAFV), and Bundibugyo virus (BDBV)^36^. There are two types of virus in the *Marburgvirus* genus: Marburg virus (MARV) and Ravn virus (RAVV)^37^. Lloviu virus (LLOV) is the only known species of the third genus *Cuevavirus*, discovered in bats in the Iberian Peninsula^36^.

**Figure 4:**
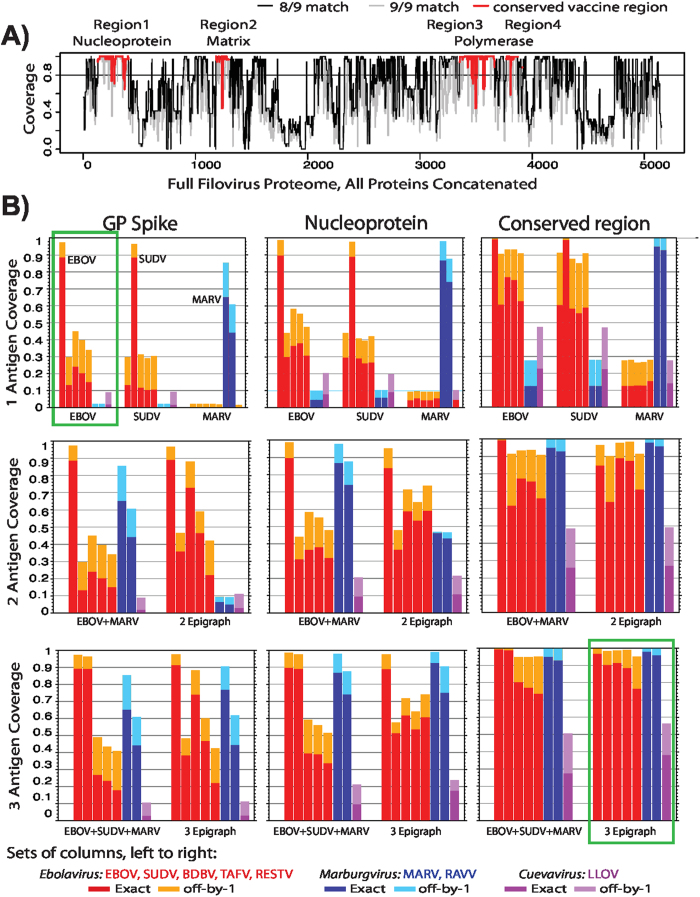
Ebola Epigraphs. **(A)** PTE Epigraph coverage of Ebola relative to a full proteome alignment, including one representative sequence per human outbreak. All 7 proteins in the Filovirus proteome (excluding soluble GPs) were concatenated, 2 Epigraph sequences were generated spanning the full proteome, and these were used to identify the most conserved regions in the proteome based on PTE coverage, highlighted in red. The black line shows 8/9 coverage, the gray line the 9/9, of the population by the 2 Epigraphs, for each consecutive 9-mer in the alignment. The four highly conserved regions together span 825 amino acids. **(B)** PTE coverage of Filovirus species by different vaccine options. The natural vaccine candidates used were the reference strains EBOV Yambuku-Mayinga, NC_002549; SUDV Gulu, NC_006432; and MARV Mt. Elgon-Musoke, NC_001608. (The four-letter uppercase species names the use standard nomenclature, described in the text). Columns represent the average PTE coverage for a given species, ordered left-to-right according to the legend, for different vaccine options. Deeper colors show 9/9 PTE matches, lighter colors the added coverage by 8/9 matches. *Ebolavirus* genus species are red, *Marburgvirus* blue, and *Cuevavirus* purple. There is a high level of PTE coverage within-species. Vaccines being evaluated in West Africa use a natural EBOV GP antigen^30^,^40^, and PTE coverage would be excellent for other EBOV strains, but poor for other species (green box, top left). In contrast, a three-antigen conserved-region Epigraph has excellent coverage across all known sequences sampled from *Filoviridae* (green box, bottom right).

Most vaccine efforts (reasonably) focus on EBOV, SUDV, and MARV, as these viruses are historically the most frequent causes of these outbreaks^30^,^38^. There is a high degree of conservation within a species (Fig. 4B), so, for example, a response to any EBOV vaccine would likely be cross-reactive with other EBOV outbreaks. Future outbreaks, however, may result from the re-emergence of a virus from a rare species, or a virus from a new species not yet encountered. There is historical precedent for this: Bundibugyo virus was first identified in a 2007 outbreak and re-emerged in 2012, and Taï Forest virus infected an individual studying a chimpanzee outbreak in 1994. Reston virus has recurrently emerged in primate facilities, is lethal in monkeys, infects pigs, and sometimes causes exposed people to develop antibodies, although to date all people who developed antibodies have been asymptomatic^34^. Thus it may be prudent to develop a vaccine that is potentially effective across all 8 distinctive *Filoviridae* species/variants^39^, in parallel with the currently prioritized development of effective vaccines against common outbreak species^40^.

We first used PTE coverage by full proteome Epigraphs as a means to define the four most conserved regions across all members of the *Filoviridae* family. This was based on a comprehensive set of full genome sequences^32^. To identify conserved regions, we created an aligned two-antigen Epigraph vaccine solution for the full filovirus proteome, and used local PTE coverage provided by the Epigraph solution as our measure of conservation. We used an 8/9 matched minimum coverage of 80% as the criterion to define conserved regions. We required a minimum contiguous stretch of at least 100 amino acids for inclusion as a conserved region, tolerating short dips in coverage due to diverse PTEs caused by isolated variable positions. Based these criteria, we identified the four most conserved regions in the proteome (Fig. 4A and Supplementary Table 6). The conserved regions collectively span 825 amino acids, which is a reasonable insert size for many vectors.

Having identified conserved regions, our next step was to design the vaccine. Because a future outbreak is most likely to be the result of a virus from one of the common species, we did not want to compromise the cross-reactive potential for those viruses. But, as discussed above, it is also possible that a future outbreak may more closely resemble rare-in-human, or even as-yet-undiscovered, members of the *Filoviridae* family. More specifically, our criteria are to:

-  Preserve PTE coverage of EBOV, the most common species in human outbreaks
-  Maintain excellent PTE coverage of SUDV and MARV, which also have caused recurrent outbreaks; and
- given the constraints imposed by the first two criteria, provide extensive PTE coverage of all other Filovirus species.

There are many possible paths to achieve good *Filoviridae* PTE coverage, and we systematically explored the outcomes of different design strategies, including optimization of Epigraph vaccine antigens using the 34 outbreak sequences simultaneously, as well as combinations that used serial optimization, either starting with a natural sequence, or starting with an Epigraph solution based on the 5 representative sequences selected from members of the *Ebolavirus* genus, or on a set of 8 representative sequences that sampled filovirus diversity. We compared PTE coverage based on full proteins that have been commonly used in vaccines, to the coverage based on conserved regions. We also explored the impact of optimization on imperfect matches, and exclusion of rare epitopes. A priori, we didn’t know which of these strategies would provide the best solutions, and in these series of comparisons, dozens of Epigraphs runs were conducted and compared; the speed of the Epigraph code enabled a thorough and systematic exploration of design options. In Supplementary Table 7, we show we show coverage results for the subset solutions that we considered of greatest interest. We show the ‘B’ and ‘E’ solutions from those tables, with coverage breakdowns for each species, in Fig. 4, as they provide the best 2 and 3 antigen solution given the specific criteria i-iii discussed above. The B solution started with a 5 species *Ebolavirus* Epigraph solution; this was fixed (we call it sequence “a”) to enforce good coverage of this historically important genus, the cause of many highly lethal outbreaks. Then a second Epigraph sequence was designed that offered maximum complementary coverage of the full 34 sequence outbreak set relative to sequence “a”, for use as a bivalent vaccine. The E solution again started with “a”, and two Epigraphs were simultaneously solved that again offered maximum complementary coverage of the full 34 sequence outbreak set relative to “a”, this time intended for use as a trivalent vaccine.

We conclude that Epigraph vaccines based on conserved regions in the Ebola proteome suggest a pan-Filoviridae vaccine may be feasible, with the potential to maintain reactivity to the recurrent outbreak strains, while extending cross-reactivity across the known diversity of filoviruses (Fig. 4), and perhaps beyond, to viruses related to the *Filoviradae* family that have not yet been encountered. In particular, a trivalent conserved-region Epigraph vaccine achieves (>90%) PTE coverage of viruses across the *Marburgvirus* and *Ebolavirus* genera, with significant cross-reactive potential against the very distinctive *Cuevavirus* (Fig. 4). In contrast, single natural EBOV Glycoprotein or Nucleoprotein vaccines^30^,^40^ have poor cross-reactive potential with viruses of other species; the multi-modality in the plots in Fig. 4 is due to the fact that within species, even between outbreaks viruses are highly related, but between species and genera sequence distances are much greater. Even combinations of natural antigens have limited potential for cross-reactivity (Fig. 4).

## Discussion

Building on the principles used for Mosaic vaccines – namely, the collective design of multiple antigens to maximize PTE coverage – Epigraphs employ a graph-based dynamic programming strategy that is computationally much more efficient and, under restricted conditions, mathematically optimal (as we show in the Methods section). This high performance at low cost expands the design space for novel vaccine approaches. Our Epigraph vaccine design tool suite^20^ includes the ability to define and exclude rare epitopes, to use aligned or unaligned input sequences, and to use inexact matches as an optimization criterion.

By maximizing PTE coverage, polyvalent Epigraphs are markedly more efficient than natural sequences in making use of the sequence space available in antigen inserts. The most common forms of epitopes are favored, while rare type-specific epitopes, which are found in virtually every natural HIV strain, are disfavored, and can be explicitly excluded. If a variant of an epitope is already present in one antigen, other antigens in the set will tend to pick up other common variants. Such epitope complementarity in Mosaic antigen sets has been shown experimentally to extend both the breadth and the depth of the vaccine response in animal models^10^,^13^,^15^. Also, Epigraphs provide a logical framework for reagent as well as vaccine design: for example, Epigraph sequences could be used as a foundation to design an optimal set of peptides to explore immune responses in a population infected by a variable pathogen, and would have an advantage over a consensus sequence as amino acid positions in an alignment are not considered in isolation, rather the frequency of local combinations of amino acids are considered.

We incorporated Epigraph code into our TTV design algorithm, to use PTE similarity as a foundation for defining clusters of viruses, an immunological perspective, rather than less relevant Hamming or phylogenetic distances. TTVs are therapeutic vaccines that could best cover the population diversity for the purpose of tailoring a vaccine to individual patient’s infecting virus within the context of manufacturing a limited and feasible number of antigens. We also explored the idea that in designing TTVs (and more generally, in choosing how many antigens to deliver in any polyvalent vaccine), the benefits of increased PTE coverage should be balanced with the cost of including mismatched (*i.e.,* extra) epitopes in a vaccine. The impact of mismatched epitopes on T-cell responses is not understood, but can now be experimentally evaluated in the context of antigen presentation in RhCMV vectors in NHPs, where the effect of varying these parameters on protective efficacy be tested experimentally.

Previous experiments in NHPs using Mosaic HIV vaccines show that use of polyvalent proteins, computationally designed to maximize PTE coverage, results in vaccine-elicited T-cell responses that have increased cross-reactivity and potency^8^,^10^,^11^,^14^,^15^. Further, blending conserved region approaches and PTE coverage design may be advantageous^6^,^16^,^25^. By analogy, coupling Epigraphs with a conserved region strategy enables vaccine designs with the potential to extend cross-reactivity across *Filoviridae*, which may be important in an uncertain future when the next outbreak virus is not predictable. For the pan-filovirus conserved region design, we used Epigraph to create a local epitope coverage map that provided an immunologically relevant, epitope-centric identification of the most conserved regions in the diverse viral family. The exploration of many possible design options enabled us to identify a vaccine design that has the potential to provide cross-reactive coverage across all of the *Filoviridae* family without compromising the coverage of ebolaviruses and marburgviruses. Of course the vaccine antigen designs we present here are based on predictions of cross-reactive potential, and have yet to be experimentally validated. But given earlier success with Mosaic vaccines in NHPs, and the extent of PTE coverage we observe, these Epigraph designs have promise, and experimental evaluation of these designs is underway. Our new Epigraph algorithm allowed us to solve design problems that were intractable using our first generation Mosaic strategy. We believe the Epigraph code has the potential to aid in discovery of optimal design strategies for other highly variable viruses.

## Methods


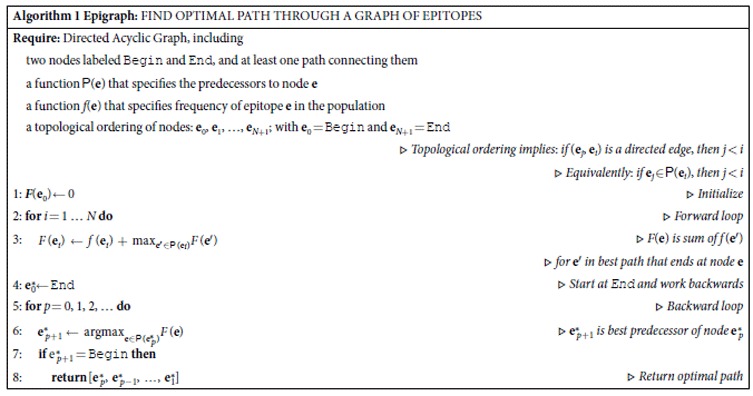


### Epigraph algorithm

A sample set 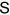 = {**s**_1_, **s**_2_, …, **s**_*N*_} of *N* protein sequences is taken to characterize the variability of a virus over a population that will be targeted for vaccine use *e.g.,* a phylogenetic clade, a country, or the world. Each PTE is assigned a frequency corresponding to the fraction of sequences in 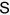 in which the PTE appears. For example, if **e** = VTSSNMNNA, and if *n* is the number of sequences in the sample set 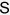 in which the 9-mer VTSSNMNNA appears, then its frequency is given by *f*(**e**) = *n*/*N*.

We will write 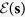 as the set of PTEs that appear in the sequence **s**. For example, if **s** = VTSSNMNNADSVWLR…, then 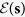 = {VTSSNMNNA, TSSNMNNAD, SSNMNNADS, SNMNNADSV, NMNNADSVW, MNNADSVWL, NNADSVWLR, …}. For a *set* 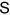 of several sequences, 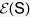 is the union of the sets 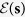 over all **s** ∈ 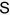; in other words, 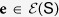 if and only if 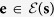 for some **s** ∈ 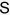. Even if an epitope **e** appears in multiple sequences, it still appears only once in 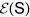.

An artificial antigen **q** is a sequence that resembles a natural protein but contains PTEs that correspond to the most frequently appearing PTEs in the population sample 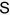. Writing 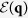 as the set of PTEs that appear in **q**, we say that an antigen **q** exhibits good coverage if the *f*(**e**)’s are large for the **e**’s in 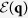. More formally, we define


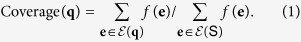


The numerator is the sum of the frequencies of PTEs that appear in **q**, and the denominator is the sum over *all* PTEs. This coverage corresponds to the total cross-reactive potential of all the epitopes in the vaccine antigens. We don’t have a detailed model for how reactive each epitope is, or even for which *k*-mers are true epitopes;


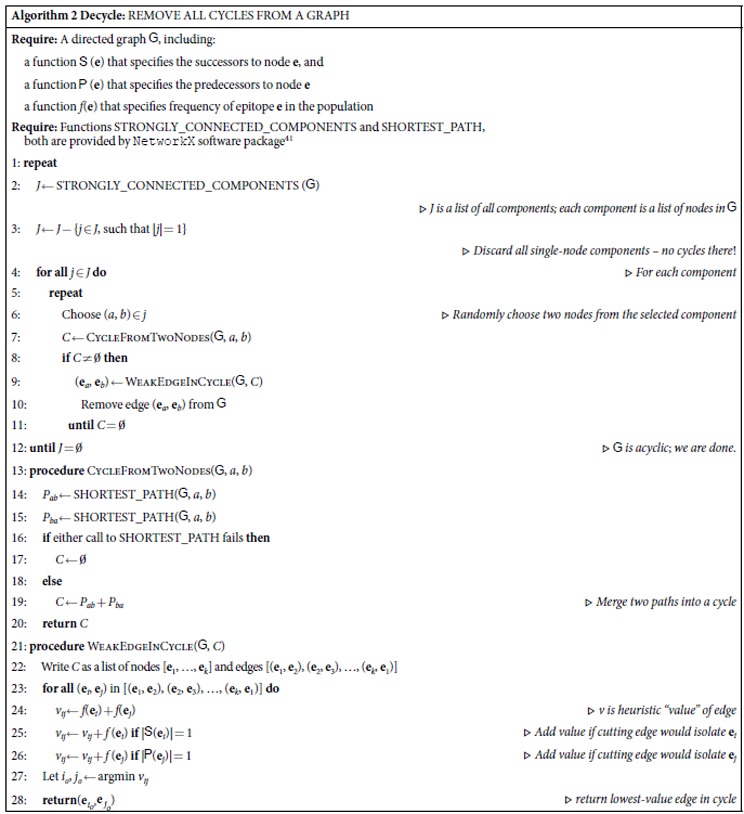



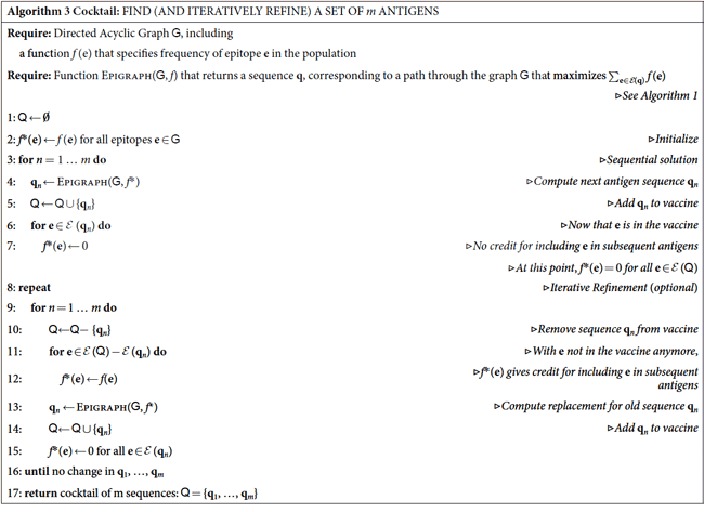



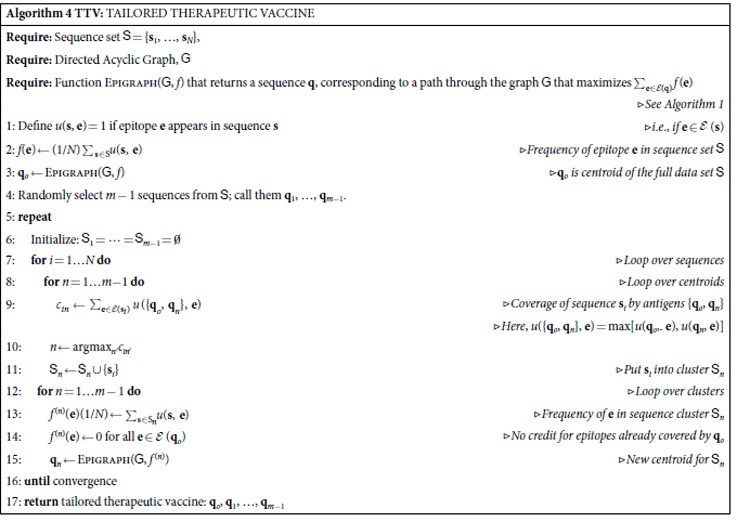


in the face of this uncertainty, we treat all *k*-mers equally. For a highly immunogenic protein like HIV-1 Gag, T-cell epitopes have been identified in the literature (and summarized in the Los Alamos HIV database^42^) that tile across the entire Gag protein, providing a rationale for this assumption.

A polyvalent vaccine consists of several artificial antigens: 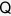 = {**q**_1_,…,**q**_*m*_}. And Coverage(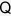) is given by Eq. (1) with the sum over 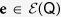.

The main idea in Epigraph is that we can express this formulation as a directed graph (Fig. 1). Each node in the graph corresponds to a distinct *k*-mer, and a directed edge connects two *k*-mers (**e**_*a*_, **e**_*b*_) if they overlap by *k* − 1 characters, as illustrated in the Fig. 1(b) inset. We remark that this *k*-mer overlap graph, which is closely related to a de Bruijn graph^43^, is widely used in genome assembly^44^,^45^.

A *path* through the graph is a connected sequence of nodes **e**_1_, **e**_2_, …, **e**_*L*_: there is a directed edge from **e**_1_ to **e**_2_, from **e**_2_ to **e**_3_, and so on until the last edge connects **e**_*L*−1_ to **e**_*L*_. Such a path corresponds to a sequence of *L* + *k* − 1 characters, which defines the artificial antigen **q**. The coverage associated with that antigen is directly proportional to the sum of the frequencies associated to the nodes in the path: *f*(**e**_1_) + *f*(**e**_2_) + 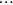 + *f*(**e**_*L*_).

For computational convenience, we add Begin and End nodes to the graph, connected respectively to the first and last *k* characters in each sequence. Epigraph (see Algorithm 1) finds a path *P* from Begin to End that optimizes the total frequency ∑_**e**∈*P*_*f*(**e**) of epitopes in that path. The algorithm for finding the optimal path is straightforwardly equivalent to well-known algorithms in graph theory^46^, and uses dynamic programming, a strategy often used in bioinformatic applications^47^,^48^. It consists of a forward loop, followed by a backward loop. The forward loop computes *F*(**e**) for all the nodes, where *F*(**e**) is the maximum total frequency over all paths that end in **e**. The backward loop builds the path that achieves the maximal score.

Let 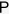
(**e**) be the set of predecessors of node **e**: that is, the set of nodes **e**′ for which there exists a directed edge that connects from **e**′ to **e**. Then we have


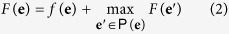


If the set of predecessors 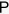
(**e**) is empty, then we define *F*(**e**) = *f*(**e**). In particular, *F*(Begin) = *f*(Begin) = 0. If all of the sequences in S contain only amino acid characters, then the Begin node will be the only node with no predecessors. If there is a non-amino-acid character (*e.g.,* an ‘X’ indicating an ambiguous base call in the DNA sequence, or a ‘#’ indicating a frame shift) in any of the sequences, then the PTE immediately after that character might also lack a predecessor. For a directed acyclic graph, there exists a “topological ordering” of the epitopes^46^, **e**_1_, **e**_2_, …, with the property that if (**e**_*i*_, **e**_*j*_) is a directed edge, then *i* < *j*. By proceeding in this topological order, we can straightforwardly evaluate Eq. (2) for all the nodes.

Having evaluated *F*(**e**) for all the nodes **e**, we will start at the node 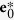 = End, and iterate backwards:


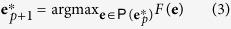


If the set 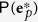 is empty, then 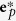 has no predecessors, and we are finished: usually, 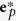 = Begin. The sequence of epitopes 
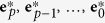
 corresponds to a reconstructed sequence **q** of *p* + *k* − 2 characters that optimizes the epitope coverage by an intact artificial protein that resembles a natural protein. If the argmax operator does not have a unique value, there are multiple solutions, all equivalent and optimal in the sense of coverage.

If this directed graph has no cycles, then Epigraph finds a path that maximizes Eq. (1), providing a rigorously optimal solution. This optimization is done with computational effort that scales only linearly with the size (as measured in nodes and edges) of the graph. In practice the directed graph created from 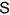 may not be acyclic, though it is often very nearly so, especially for larger values of *k*. For this case, we developed a heuristic scheme to “de-cycle” the graph, by iteratively identifying cycles and then removing low-value edges until no cycles remain (see Algorithm 2 and Methods: De-cycling).

As an aside, we further remark that the logic that defines *F*(**e**) in Eq. (2) can be employed to define *x*(**e**) for all epitopes in the graph:


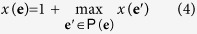


If (**e**_*a*_, **e**_*b*_) is a directed edge in the graph, then Eq. (4) guarantees that *x*(**e**_*a*_) < *x*(**e**_*b*_). In particular, if we use *x*(**e**) as a horizontal position associated with node **e** (which we do in Fig. 1), then we will have the property that all directed edges point from left to right. As with Eq. (2), the definition in Eq. (4) requires that the graph be acyclic.

If a user wishes to exclude rare epitopes, they an do this by selecting a cutoff frequency for exclusion for an Epigraph run. The Epigraph tool suite enables a user to explore of the cost in terms of overall coverage as the cutoff frequency increases (Fig. 2), to make an informed decision regarding the selection of the cutoff value. Epigraph will then eliminating nodes in the graph for which *f*(**e**) ≤ *f*_*o*_ = *n*_*o*_/*N*, where *f*_*o*_ is a cutoff frequency (and *n*_*o*_ is a cutoff count), and *N* is the number of sequences in the population.

### De-cycling

The population of sequences gives rise to a directed graph, but this graph may contain cycles, and the Epigraph algorithm requires that the graph be acyclic. One way cycles can arise is when an identical *k*-mer is found directly repeated in the same sequence; this is not common, but it does happen. In the Los Alamos HIV database 2014 alignment, out of *N* = 4250 HIV Env sequences, 91 of them (2.1%) have at least one 9-mer directly repeated in a sequence. Similarly 0.3% of Nef, 0.8% of Pol, and 1.3% of Gag sequences, carry such repeats. A more common way for cycles to arise, however, comes from effective repeats of an epitope across multiple sequences.

On the other hand, we have found that, particularly for larger values of *k* (and larger values of *f*_*o*_), the graph is often very nearly acyclic, and can be made acyclic with only a few perturbations to the graph. The optimal solution to this perturbed graph is then taken as a nearly-optimal solution to the original graph. The problem of removing the least number of edges to produce an acyclic graph is equivalent to the NP-hard “minimum feedback arc set” problem^49^,^50^. Thus we use a heuristic approach for making these perturbations; we keep the same nodes from the original graph, but successively cut edges until an acyclic graph remains.

To eliminate cycles, we first have to find cycles, and to help with this task, we decompose the graph into “strongly connected components” (for an acyclic graph, every node is its own strongly connected component) – a task that can be performed in linear time^51^. Within a single strongly connected component, there exists a path from every node to every other node, and this makes cycles easy to find: if **e**_*a*_ and **e**_*b*_ are two nodes in the same component, then the directed path from **e**_*a*_ to **e**_*b*_ can be merged with the directed path from **e**_*b*_ to **e**_*a*_ to form a cycle that includes both **e**_*a*_ and **e**_*b*_.

Each time a cycle is located in the graph, we choose one of the edges in the cycle to remove from the graph. This choice is heuristic, but since cutting edges has the effect of isolating nodes, we seek cuts that isolate low-value nodes. For each edge (**e**_*a*_, **e**_*b*_) we define a value, based on *f*(**e**_*a*_) and *f*(**e**_*b*_); then we choose the edge with the smallest value and remove it from the graph. A very simple and (empirically) effective heuristic is to take the value to be the sum *f*(**e**_*a*_) + *f*(**e**_*b*_). In our experiments, we employed a slight modification of this heuristic. If **e**_*a*_ is the *sole* predecessor of **e**_*b*_, then cutting edge (**e**_*a*_, **e**_*b*_) will isolate node **e**_*b*_, so we add a further cost of *f*(**e**_*b*_). Similarly, if **e**_*b*_ is the sole successor to **e**_*a*_, then we add *f*(**e**_*a*_). See Algorithm 2 for details.

### Polyvalent vaccines

In the polyvalent, or “cocktail”, version of the problem, we seek *m* > 1 artificial sequences 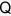 = {**q**_1_, …, **q**_*m*_} that collectively maximize the coverage of PTEs in the sample target population 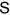. Typically *m* is small, only 2 or 3, due to both implementation costs and the biological “cost” of including more rare epitopes as *m* increases, which may divert the immune response from more useful conserved epitopes. We write 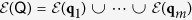 as the set of epitopes that appear in at least one of the sequences in 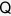, and seek to optimize the sum 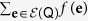 of the frequencies for all the epitopes that appear in 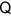.

To find a cocktail of *m* > 1 antigens, the Epigraph algorithm is applied in a sequential manner, as shown in Algorithm 3. To see how this works, suppose we have a solution to the *m*′-sequence problem; to extend this to the (*m*′ + 1)-sequence problem, we try to optimize the *complementary* coverage, and pick up to the extent possible high-frequency PTEs that were not sampled in the first *m*′ sequences. This is done by setting the *f* value of the already-covered PTEs to zero. The graph structure is the same, but the update of the cumulative scores is based on these new *f* values. Although revisiting epitopes from the {**q**_1_, …, **q**_*m*′_} sequences is allowed if essential to complete the path, it is discouraged because there is no gain in the coverage score. Specifically, define the modified frequency


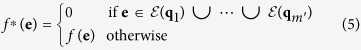


and, as in Eq. (2), let 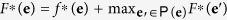.

Thus, for instance, we can find an *m*′ = 1 solution using Epigraph on the original frequencies *f*(**e**); then extend to *m*′ = 2, 3, …, *m* by optimizing complementary coverage at each stage, using the modified f*(**e**). The *m* = 1 + 1 column in Supplementary Table 2 corresponds to this sequential approach.

Once an initial polyvalent solution has been determined, iterative refinement of sequential solutions can improve the final coverage. Given initial sequences **q**_1_, **q**_2_, …, **q**_*m*_, we can go back and recompute a new solution for **q**_1_. This is done by starting with the original frequency values for each of the epitopes, but setting to zero those epitopes that are covered by **q**_2_, …, **q**_*m*_. The optimization of this complementary coverage problem leads to a new **q**_1_. One can loop through all of the initial solutions this way, each time optimizing the appropriate complementary coverage.

The iterative refinement scheme can also be applied to other initial conditions; *e.g.,* one can use a consensus, natural or Mosaic solution as an initial sequence. Supplementary Table 4 shows how a Mosaic solution can be improved by using the Mosaic solution as a starting place for an iterative Epigraph refinement. If Mosaic antigens are allowed to evolve for many generations, they may eventually evolve to a solution that is better in terms of PTE coverage than a first-pass Epigraph solution. But even these solutions might be improved with iterative Epigraph refinement.

Multiple trials can also be used to improve coverage. Here, instead of using Epigraph to obtain an *m*′ = 1 solution, use a random sequence for **q**_1_. With this as a starting point, sequentially add new sequences **q**_2_, …, **q**_*m*_, followed by iterative refinement until convergence is achieved. We can do this for many random initial sequences, and keep the solution that gives the best coverage. Iterative refinements with multiple trials were used for the *m* = 2 column in Supplementary Table 2.

## Additional Information

**How to cite this article**: Theiler, J. *et al*. Epigraph: A Vaccine Design Tool Applied to an HIV Therapeutic Vaccine and a Pan-Filovirus Vaccine. *Sci. Rep.* **6**, 33987; doi: 10.1038/srep33987 (2016).

## Supplementary Material

Supplementary Information

## Figures and Tables

**Table 1:** 

**Algorithm 1 Epigraph:** find optimal path through a graph of epitopes	
**Require:** Directed Acyclic Graph, including	
two nodes labeled Begin and End, and at least one path connecting them	
a function 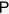 (**e**) that specifies the predecessors to node **e**	
a function *f*(**e**) that specifies frequency of epitope **e** in the population	
a topological ordering of nodes: **e**_0_, **e**_1_, …, **e**_*N*+1_; with **e**_0_ = Begin and **e**_*N*+1_ = End	
	▹ *Topological ordering implies*: *if* (**e**_*j*_, **e**_*i*_) *is a directed edge*, *then* *j* < *i*
	▹ *Equivalently*: *if* **e**_*j*_ ∈ 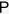 (**e**_*i*_), *then* *j* < *i*
1: ***F***(**e**_0_) ← 0	▹ *Initialize*
2: **for** *i* = 1 … *N* **do**	▹ *Forward loop*
3: 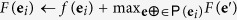	▹ *F*(**e**) *is sum of f* (**e**′)
	▹ *for* **e**′ *in best path that ends at node* **e**
4: 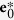 ← End	▹ *Start at* *End* *and work backwards*
5: **for** *p* = 0, 1, 2, … **do**	▹ *Backward loop*
6: 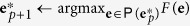	▹ 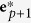 *is best predecessor of node* 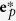
7: **if** 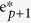 = Begin **then**	
8: **return** 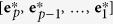	▹ *Return optimal path*

**Table 2:** 

**Algorithm 2 Decycle:** REMOVE ALL CYCLES FROM A GRAPH	
**Require:** A directed graph 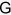 , including:	
a function 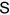 (**e**) that specifies the successors to node **e**, and	
a function 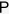 (**e**) that specifies the predecessors to node **e**	
a function *f*(**e**) that specifies frequency of epitope **e** in the population	
**Require:** Functions STRONGLY_CONNECTED_COMPONENTS and SHORTEST_PATH, both are provided by NetworkX software package^41^	
1: **repeat**	
2: *J* ← STRONGLY_CONNECTED_COMPONENTS ( 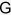 )	
	▹ *J is a list of all components; each component is a list of nodes in* 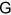
3: *J* ← *J* − {*j* ∈ *J*, such that |*j*| = 1}	
	▹ *Discard all single-node components – no cycles there*!
4: **for all** *j* ∈ *J* **do**	▹ *For each component*
5: **repeat**	
6: Choose (*a*, *b*) ∈ *j*	▹ *Randomly choose two nodes from the selected component*
7: *C* ← CYCLEFROMTWONODES( 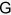 , *a*, *b*)	
8: **if** *C* ≠ ∅ **then**	
9: (**e**_*a*_, **e**_*b*_) ← WEAKEDGEINCYCLE( 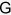 , *C*)	
10: Remove edge (**e**_*a*_, **e**_*b*_) from 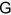	
11: **until** *C* = ∅	
12: **until** *J* = ∅	▹ 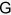 *is acyclic*; *we are done*.
13: **procedure** CYCLEFROMTWONODES( 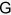 , *a*, *b*)	
14: *P*_*ab*_ ← SHORTEST_PATH( 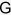 , *a*, *b*)	
15: *P*_*ba*_ ← SHORTEST_PATH( 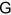 , *a*, *b*)	
16: **if** either call to SHORTEST_PATH fails **then**	
17: *C* ← ∅	
18: **else**	
19: *C* ← *P*_*ab*_ + *P*_*ba*_	▹ *Merge two paths into a cycle*
20: **return** *C*	
21: **procedure** WEAKEDGEINCYCLE( 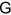 , *C*)	
22: Write *C* as a list of nodes [**e**_1_, …, **e**_*k*_] and edges [(**e**_1_, **e**_2_), (**e**_2_, **e**_3_), …, (**e**_*k*_, **e**_1_)]	
23: **for all** (**e**_*i*_, **e**_*j*_) in [(**e**_1_, **e**_2_), (**e**_2_, **e**_3_), …, (**e**_*k*_, **e**_1_)] **do**	
24: *v*_*ij*_ ← *f*(**e**_*i*_) + *f*(**e**_*j*_)	▹ *v is heuristic “value” of edge*
25: *v*_*ij*_ ← *v*_*ij*_ + *f* (**e**_*i*_) **if** | 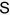 (**e**_*i*_)| = 1	▹ *Add value if cutting edge would isolate* **e**_*i*_
26: *v*_*ij*_ ← *v*_*ij*_ + *f* (**e**_*j*_) **if** | 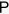 (**e**_*j*_)| = 1	▹ *Add value if cutting edge would isolate* **e**_*j*_
27: Let *i*_*o*_, *j*_*o*_ ← argmin *v*_*ij*_	
28: **return**( 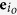 , 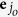 )	▹ *return lowest-value edge in cycle*

**Algorithm 3 Cocktail:** FIND (AND ITERATIVELY REFINE) A SET OF *m* ANTIGENS	
**Require:** Directed Acyclic Graph 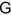 , including	
a function *f* (e) that specifies frequency of epitope **e** in the population	
**Require:** Function EPIGRAPH( 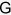 , *f*) that returns a sequence **q**, corresponding to a path through the graph 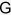 that maximizes 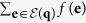	
▷*See Algorithm 1*	
1: 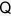 ← ∅	
2: f*(**e**) ← *f* (**e**) for all epitopes **e** ∈ 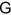	▷*Initialize*
3: **for** *n* = 1 … *m* **do**	▷*Sequential solution*
4: **q**_*n*_ ← EPIGRAPH( 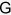 , f*)	▷*Compute next antigen sequence* **q**_*n*_
5: 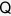 ← 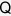 ∪ {**q**_*n*_}	▷*Add* **q**_*n*_ *to vaccine*
6: **for e** ∈ 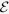 (**q**_*n*_) **do**	▷*Now that* **e** *is in the vaccine*
7: f*(**e**) ← 0	▷*No credit for including* **e** *in subsequent antigens*
	▷*At this point*, f*(**e**) = 0 *for all* **e** ∈ 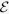 ( 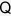 )
8: **repeat**	▷*Iterative Refinement* (*optional*)
9: **for** *n* = 1 … *m* **do**	
10: 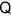 ← 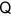 − {**q**_*n*_}	▷*Remove sequence* **q**_*n*_ *from vaccine*
11: **for e** ∈ 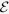 ( 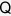 ) − 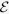 (**q**_*n*_) **do**	▷*With* **e** *not in the vaccine anymore*,
12: f*(**e**) ← *f*(**e**)	▷f*(**e**) *gives credit for including* **e** *in subsequent antigens*
13: **q**_*n*_ ← EPIGRAPH( 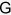 , f*)	▷*Compute replacement for old sequence* **q**_*n*_
14: 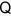 ← 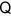 ∪ {**q**_*n*_}	▷*Add* **q**_*n*_ *to vaccine*
15: f*(**e**) ← 0 for all **e** ∈ 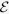 (**q**_*n*_)	
16: **until** no change in **q**_1_, …, **q**_*m*_	
17: **return** cocktail of m sequences: 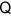 = {**q**_1_, …, **q**_*m*_}	

**Algorithm 4 TTV:** TAILORED THERAPEUTIC VACCINE	
**Require:** Sequence set 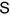 = {**s**_1_, …, **s**_*N*_},	
**Require:** Directed Acyclic Graph, 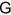	
**Require:** Function EPIGRAPH( 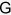 , *f*) that returns a sequence **q**, corresponding to a path through the graph 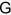 that maximizes 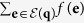 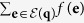	
	▷*See Algorithm 1*
1: Define *u*(**s**, **e**) = 1 if epitope **e** appears in sequence **s**	▷*i*.*e*., *if* **e** ∈ 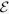 (**s**)
2: *f*(**e**) ← (1/*N*) 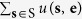	▷*Frequency of epitope* **e** *in sequence set* 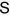
3: **q**_*o*_ ← EPIGRAPH( 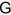 , *f*)	▷**q**_*o*_ *is centroid of the full data set* 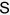
4: Randomly select *m* − 1 sequences from 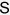 ; call them **q**_1_, …, **q**_*m*−1_.	
5: **repeat**	
6: Initialize: 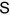 _1_ = … = 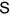 _*m*−1_ = ∅	
7: **for** *i* = 1…*N* **do**	▷*Loop over sequences*
8: **for** *n* = 1…*m*−1 **do**	▷*Loop over centroids*
9: 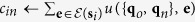	▷*Coverage of sequence* **s**_*i*_ *by antigens* {**q**_*o*_, **q**_*n*_}
	▷*Here*, *u*({**q**_*o*_, **q**_*n*_}, **e**) = max[*u*(**q**_*o*_,. **e**), *u*(**q**_*n*_, **e**)]
10: *n* ← argmax_*n*′_*c*_*in*′_	
11: 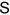 _*n*_ ← 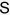 _*n*_ ∪ {**s**_*i*_}	▷*Put* **s**_*i*_ *into cluster* 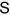 _*n*_
12: **for** *n* = 1…*m* − 1 **do**	▷*Loop over clusters*
13: *f*^ (*n*)^(**e**)(1/*N*) ← 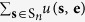	▷*Frequency of* **e** *in sequence cluster* 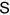 _*n*_
14: *f*^ (*n*)^(**e**) ← 0 for all **e** ∈ 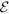 (**q**_*o*_)	▷*No credit for epitopes already covered by* **q**_*o*_
15: **q**_*n*_ ← EPIGRAPH( 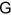 , *f*^ (*n*)^)	▷*New centroid for* 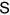 _*n*_
16: **until** convergence	
17: **return** tailored therapeutic vaccine: **q**_*o*_, **q**_1_, …, **q**_*m*−1_	
