# Supplementary material for: Epigraph: A Vaccine Design Tool Applied to an HIV Therapeutic Vaccine and a Pan-Filovirus Vaccine
Source: Sci Rep. 2016 Oct 5;6:33987. doi: 10.1038/srep33987 (PMC5050445; doi:10.1038/srep33987)
Supplement: Supplementary Information [file srep33987-s1.pdf]

Supplementary Material

Epigraph: A Vaccine Design Tool Applied to an HIV Therapeutic Vaccine and a Pan-Filovirus Vaccine

James Theiler[1,2], Hyejin Yoon[1], Karina Yusim[1,2], Louis J. Picker[3], Klaus Frueh[3], and Bette Korber[1,2]

[1]Los Alamos National Laboratory, Los Alamos, NM 87545, USA
[2]New Mexico Consortium, Los Alamos, NM 87544, USA
[3]Oregon Health and Science University, Portland, OR 97239, USA

Aligned Sequences

For aligned sequences, the positions $t = 1 \ldots T$ are well-defined for each amino acid character, and the artificial sequence $\mathbf{q}$ that we wish to construct will also be of length T. We write $\mathbf{s}_n[t]$ as the t'th character in the n'th sequence. It will also be useful to introduce the notation $\mathbf{s}[t:u]$ for the subsequence of $\mathbf{s}$ that begins at position t and ends at position $u-1$. We define a new frequency function that depends on position: $f(t,\mathbf{e})$ is the fraction of sequences in S for which $\mathbf{e} = \mathbf{s}[t:t+k]$. The "coverage" of a sequence $\mathbf{q}$ is given by

$$\frac{1}{T-k}\sum_{t=1}^{T-k} f(t,\mathbf{q}[t:t+k]). \tag{S-1}$$

For the aligned-sequence problem, the nodes of our graph will be associated with distinct $(t,\mathbf{e})$ values.

In order to align sequences, one has to deal with insertions and deletions, and this introduces gaps into the aligned sequences. For example, the sequences `ACDEGHI` and `ADEFGHI` are better aligned as `ACDE-GHI` and `A-DEFGHI`. The gap character is treated differently from an amino acid character:

1. For a given sequence $\mathbf{s}$, if $\mathbf{s}[t]$ is not a gap character, then we associate the epitope at position t as the first k non-gap characters, beginning with the character $\mathbf{s}[t]$. For example if $k = 9$ and $\mathbf{s}$ =`GNF--RNQRK-IVKCFNCGK...`, then the PTE associated with $t = 2$ is `NFRNQRKIV`.

2. If $\mathbf{s}[t]$ is the gap character, then we make a "placeholder epitope" whose first character is the gap character, and whose subsequent characters are the next $k-1$ non-gap characters. For these epitopes, we set $f(\mathbf{e}) = 0$. In the example above, the PTE associated with $t = 4$ is the placeholder epitope `-RNQRKIVK`.

The rules for connecting edges to consistent epitope pairs are also modified. The first rule is that edges are only supplied for adjacent positions; a directed edge can only connect a node at position t with another at position $t+1$. As with ungapped sequences, two adjacent epitopes are considered consistent if the last $k-1$ characters of the first epitope agree with the first $k-1$ characters of the second epitope. But we also consider the pair consistent if the second epitope begins with a gap character, and the remaining $k-1$ characters match the last $k-1$ characters of the first epitope. For example: `ACDEFGHIK` and `-CDEFGHIK` are consistent, `-CDEFGHIK` is consistent with itself; and `-CDEFGHIK` and `CDEFGHIKL` are consistent.

Inexact matches

To evaluate the inexact-match coverage, we introduce a set $\mathsf{H}_d(\mathbf{e})$ that includes all epitopes within Hamming distance d of the epitope $\mathbf{e}$. If $\mathscr{E}$ is a set of epitopes, then we write $\mathsf{H}_d(\mathscr{E})$ as the set of all epitopes that are within distance d of some epitope in $\mathscr{E}$. That is, $\mathsf{H}_d(\mathscr{E}) = \bigcup_{\mathbf{e}\in\mathscr{E}} \mathsf{H}_d(\mathbf{e})$. Similar to Eq. (1), we can define the inexact-match coverage in terms of the frequencies $f(\mathbf{e})$ of all the epitopes that approximately match the epitopes in the vaccine:

$$\text{Coverage}_d(\mathbf{q}) = \sum_{\mathbf{e}\in\mathsf{H}_d(\mathscr{E}(\mathbf{q}))} f(\mathbf{e}) \Big/ \sum_{\mathbf{e}\in\mathscr{E}(\mathsf{S})} f(\mathbf{e}) \tag{S-2}$$

Note that *if* the $\mathsf{H}_d(\mathbf{e})$ were disjoint for all $\mathbf{e}\in\mathscr{E}$, then we would be able to write

$$\sum_{\mathbf{e}\in\mathsf{H}_d(\mathscr{E})} f(\mathbf{e}) = \sum_{\mathbf{e}\in\mathscr{E}}\sum_{\mathbf{e}'\in\mathsf{H}_d(\mathbf{e})} f(\mathbf{e}') = \sum_{\mathbf{e}\in\mathscr{E}} \widetilde{f}(\mathbf{e}) \tag{S-3}$$

where

$$\widetilde{f}(\mathbf{e}) = \sum_{\mathbf{e}'\in\mathsf{H}_d(\mathbf{e})} f(\mathbf{e}'), \tag{S-4}$$

which suggests that optimization based on $\widetilde{f}(\mathbf{e})$, in place of $f(\mathbf{e})$, would optimize off-by-d coverage. Unfortunately, however, the $\mathsf{H}_d(\mathbf{e})$ are *not* in general disjoint, so $\widetilde{f}(\mathbf{e})$ in general overestimates the "value" of $\mathbf{e}$ (*i.e.,* the contribution of $\mathbf{e}$ to the coverage). This is what prevents us from using this scheme to optimize the inexact-match coverage for unaligned sequences.

The same problem holds, in principle, for aligned sequences, but the overlap of the Hamming sets is much smaller, and we find that we *can* use this scheme for aligned sequences. We follow the idea in Eq. (S-4), and define $\widetilde{f}(t,\mathbf{e}) = \sum_{\mathbf{e}'\in\mathsf{H}_d(\mathbf{e})} f(t,\mathbf{e}')$. In our experiments, we actually used a slight variant of this expression

$$\widetilde{f}(t,\mathbf{e}) = \lambda f(t,\mathbf{e}) + \sum_{\mathbf{e}'\in\mathsf{H}_d(\mathbf{e})} f(t,\mathbf{e}') \tag{S-5}$$

where $\lambda = 0.1$. This mostly optimizes the number of inexact matches, but the $\lambda f(t,\mathbf{e})$ term provides a small bonus for exact matches.

The notion extends straightforwardly for polyvalent vaccines, though the bookkeeping is a little trickier. If $\mathscr{E}_o$ is the set of epitopes exactly matched by the first m' antigens in a vaccine, then $\mathsf{H}_d(\mathscr{E}_o)$ is the set of epitopes that are covered in an off-by-d sense. To account for the fact that these epitopes are already covered, they need to be excluded from the sum that defines $\widetilde{f}(t,\mathbf{e})$. In particular, write $\mathsf{H}'(\mathbf{e})$ as the set of epitopes that are in $\mathsf{H}_d(\mathbf{e})$ but not in $\mathsf{H}_d(\mathscr{E}_o)$. Then

$$\widetilde{f}(t,\mathbf{e}) = \begin{cases} 0 & \text{if } \mathbf{e}\in\mathscr{E}_o \\ \lambda f(t,\mathbf{e}) + \sum_{\mathbf{e}'\in\mathsf{H}'(\mathbf{e})} f(t,\mathbf{e}') & \text{otherwise,} \end{cases} \tag{S-6}$$

and the next antigen $(m'+1)$ is obtained by finding the path through the graph that optimizes $\sum_t \widetilde{f}(t,\mathbf{e}_t)$.

Filovirus alignment

We created a master input sequence alignment, using the Los Alamos Filovirus database[33]. This alignment includes 34 sequences – a single representative sequence for every human outbreak that has at least one full length genomic sequence available, as well as a representative of RESTV and LLOV, which have not been isolated from humans – to capture the extent of known *Filoviridae* diversity, while weighting the sampling towards recurrent outbreak strains. Hence the alignment includes 10 EBOV sequences, 7 SUDV, 2 BDBV, 1 TAFV, 1 RESTV, 1 LLOV, 3 RAVV, and 9 MARV. Within human outbreaks, sequences are highly similar, and so each outbreak is represented only once. To select a single representative that approximated the index case of each outbreak, we chose a sequence from the earliest sample in outbreak, when temporal data was available. If multiple isolates were sequenced from that sample, we picked a natural sequence that was either identical or closest to the consensus from the first time point. We then translated each gene, including the full-length Glycoprotein GP, but not the secreted forms, sGP and ssGP, and we concatenated the proteins into a full proteome alignment of all 7 Filovirus proteins. This served as a baseline for vaccine design. We then created two subsets of this data for staged vaccine design exploration. The first included only 8 sequences, one representative each from EBOV, SUDV, BDBV, TAFV, RESTV, LLOV, RAVV, MARV, so we could explore the vaccine design outcome if all species were weighted equally. The second one contained only a single representative virus from each of the 5 species in the *Ebolavirus* genus.

Table 1. Variation in coverage with length of epitope. Five separate Epigraph $m = 2$ solutions (with no iterative refinement or multiple starts) are computed for the B-clade Gag protein sequences. Each solution optimizes a different length epitope, with k_o varying from 8 to 12. The coverage for each solution is evaluated five different ways, based on epitope lengths varying from 8 to 12. Numbers within a column are meant to be compared, since they are all evaluated by the same criterion. We see in each case that the optimal coverage, as evaluated for k-mer epitopes, is achieved by the solution for which $k_o = k$. But we also see that the differences are not substantial.

Optimized with	Evaluated with epitope length k				
epitope length k_o	$k=8$	$k=9$	$k=10$	$k=11$	$k=12$
$k_o=8$	0.760252	0.725588	0.687727	0.650896	0.617354
$k_o=9$	0.756754	0.726877	0.692048	0.657789	0.626116
$k_o=10$	0.750047	0.723911	0.697076	0.668338	0.641497
$k_o=11$	0.749303	0.723031	0.696266	0.670036	0.644357
$k_o=12$	0.747089	0.721145	0.694929	0.669224	0.644787

Table 2. Compare 9-mer coverage for Mosaic and Epigraph. Epigraph results are based on five runs with different random number seeds; the best and the median coverage fractions are reported. (Epigraph is much faster to run than Mosaic, so it is feasible to run it five times and keep the best solution.) The main thing to notice is that the coverage fractions are so similar, with the difference typically in the fourth decimal place. Mosaics were generated using 10 hour run times on a 48 Core AMD Opteron cluster via the HIV database portal, and used population sizes of 400 (http://www.hiv.lanl.gov/content/sequence/MOSAIC/). Epigraph usually has the slight advantage; in only 7 of the 48 cases did the Mosaic solution have the best coverage. Even the median Epigraph solution outperformed Mosaic most of the time. Here, $m = 1 + 1$ corresponds to the sequential cocktail solution (optimize for $m = 1$, fix that solution, and find the optimal complementary sequence), and $m = 2$ refers to iterative refinement and multiple random starts.

	$m = 1$		$m = 1 + 1$		$m = 2$	
Protein and Clade	Mosaic	Epigraph (best/median)	Mosaic	Epigraph (best/median)	Mosaic	Epigraph (best/median)
Gag E	0.687065	0.687068<br>0.687068	0.769795	0.770351<br>0.770351	0.770029	0.770971<br>0.770971
Gag B	0.613677	0.613664<br>0.613491	0.677744	0.727352<br>0.727084	0.726545	0.727352<br>0.727091
Gag C	0.603501	0.605896<br>0.603367	0.712061	0.711849<br>0.711297	0.711460	0.711904<br>0.711813
Gag M	0.464367	0.464368<br>0.464157	0.621413	0.621209<br>0.620641	0.620995	0.621718<br>0.621405
Nef E	0.456775	0.456775<br>0.456775	0.597314	0.596507<br>0.596507	0.595638	0.596507<br>0.596507
Nef B	0.363338	0.363365<br>0.363338	0.480749	0.480515<br>0.480489	0.482010	0.482288<br>0.482001
Nef C	0.391395	0.390911<br>0.390911	0.527219	0.527535<br>0.527535	0.527125	0.527750<br>0.527750
Nef M	0.285417	0.285613<br>0.285584	0.396963	0.397462<br>0.396834	0.398129	0.398710<br>0.398410
Pol E	0.791086	0.791086<br>0.791086	0.866294	0.866522<br>0.866522	0.865880	0.866639<br>0.866639
Pol B	0.703716	0.703722<br>0.703722	0.798170	0.798906<br>0.798906	0.798047	0.798945<br>0.798906
Pol C	0.732098	0.731738<br>0.731726	0.817086	0.817760<br>0.817636	0.817081	0.817802<br>0.817636
Pol M	0.612655	0.612701<br>0.612558	0.734066	0.734081<br>0.734062	0.735310	0.735433<br>0.735260
Env B	0.378914	0.379337<br>0.379334	0.467372	0.468104<br>0.468086	0.467647	0.468269<br>0.468251
Env C	0.397118	0.397204<br>0.397204	0.489801	0.490135<br>0.490135	0.489693	0.490498<br>0.490454
Env E	0.493367	0.493401<br>0.493401	0.577307	0.577352<br>0.577352	0.576875	0.577570<br>0.577467
Env M	0.282000	0.282412<br>0.282244	0.379683	0.379903<br>0.379740	0.381089	0.381442<br>0.380971

Table 3. Aligned-sequences Epigraph performance. Performance of Epigraph applied to aligned sequences vs Mosaic for 9-mers. Because the algorithm is more nearly deterministic for the aligned case, the Epigraph results are based on a single run. As in Supplementary Table 2, note that the Epigraph and Mosaic coverage fractions very similar. But while the unaligned Epigraph usually outperformed the Mosaic solutions, the aligned Epigraph is outperformed by Mosaic just over half of the time (27 out of 48). Asterisks indicate the higher score of Mosaic vs Epigraph.

Protein and Clade	$m = 1$		$m = 1+1$		$m = 2$	
	Mosaic	Epigraph	Mosaic	Epigraph	Mosaic	Epigraph
Gag E	0.687065	0.687068*	0.769795	0.769894*	0.770029	0.770523*
Gag B	0.613677*	0.613473	0.677744	0.726896*	0.726545	0.726903*
Gag C	0.603501*	0.602685	0.712061*	0.711228	0.711460*	0.711259
Gag M	0.464367	0.465539*	0.621413*	0.619661	0.620995*	0.620100
Nef E	0.456775*	0.450319	0.597314*	0.586679	0.595638*	0.586741
Nef B	0.363338*	0.362313	0.480749*	0.477803	0.482010*	0.479306
Nef C	0.391395*	0.384473	0.527219*	0.517049	0.527125*	0.517263
Nef M	0.285417*	0.279744	0.396963*	0.387946	0.398129*	0.388959
Pol E	0.791086	0.791109*	0.866294	0.866443*	0.865880	0.866493*
Pol B	0.703716	0.703968*	0.798170	0.798931*	0.798047	0.798931*
Pol C	0.732098*	0.731857	0.817086	0.817685*	0.817081	0.817685*
Pol M	0.612655*	0.612590	0.734066	0.734103*	0.735310	0.735691*
Env B	0.378914	0.379268*	0.467372	0.467930*	0.467647	0.467930*
Env C	0.397118	0.397142*	0.489801*	0.489724	0.489693	0.489988*
Env E	0.493367*	0.493353	0.577307*	0.576248	0.576875*	0.576248
Env M	0.282000*	0.281127	0.379683*	0.377815	0.381089*	0.379208

Table 4. Improving Mosaic solutions. Epigraph can be used to "tweak" the Mosaic solution provided by the genetic algorithm; this provides a small, but always positive, improvement.

Protein and Clade	$m = 2$		
	Mosaic	Epigraph Tweak	Improvement
Gag E	0.770029	0.770501	0.000472
Gag B	0.726545	0.726733	0.000188
Gag C	0.711460	0.711603	0.000143
Gag M	0.620995	0.621332	0.000337
Nef E	0.595638	0.595845	0.000207
Nef B	0.482010	0.482053	0.000043
Nef C	0.527125	0.527776	0.000651
Nef M	0.398129	0.398636	0.000507
Pol E	0.865880	0.866402	0.000522
Pol B	0.798047	0.798393	0.000346
Pol C	0.817081	0.817775	0.000694
Pol M	0.735310	0.735427	0.000117
Env B	0.467647	0.467842	0.000195
Env C	0.489693	0.490032	0.000339
Env E	0.576875	0.577013	0.000138
Env M	0.381089	0.381510	0.000421

Table 5. Summary statistics for Tailored Therapeutic Vaccines. Shown are PTE Coverage (fraction of 9-mers in a natural strain that were perfectly matched by a 9-mers in the vaccine), and cost in terms of Extras (9 mers in the vaccine that are not found in the natural strains). In (a), these values calculated for each of 189 sequences included in the post-2005 US B clade alignment; in (b) for a set of 199 post-2005 C clade sequence from southern Africa; and in (c) a larger set of 4596 sequences was used, spanning the full M group. In these vaccine designs, n is the number of antigens delivered and m is the number of antigens manufactured (and from which the best n of m were chosen for each sequence, individually). For the straight Epigraph vaccines, $n = m$. For the Tailored vaccines, we propose manufacturing $m = 6$ antigens, and delivering $n = 2$ or $n = 3$. Coverage increases with increasing n or m, but the number of Extras depends mostly only on n. We also observed that using the conserved p24 instead of the full Gag protein dramatically increases the coverage and the reduces the number of "Extras", but p24 spans only 231 amino acids long out of Gags full 500 amino acids (based on the HIV reference strain HXB2), reducing the number of potential epitopes that could be targeted by over half.

(a) Evaluated against B-US clade

			Gag		p24	
n	*m*	Vaccine	Coverage	Extras	Coverage	Extras
1	1	M Epigraph	0.55488	237.815	0.73131	59.979
1	1	B Epigraph	0.61174	195.720	0.76421	52.646
2	2	M Epigraph	0.67323	629.333	0.84204	214.296
2	2	B Epigraph	0.72471	553.899	0.87373	201.233
3	3	B Epigraph	0.75734	786.772	0.90810	297.571
2	6	B Tailored	0.75226	536.619	0.90859	187.889
3	6	B Tailored	0.78669	773.000	0.93556	280.704

(b) Evaluated against C-SA group

			Gag		p24	
n	*m*	Vaccine	Coverage	Extras	Coverage	Extras
1	1	M Epigraph	0.39886	318.437	0.48548	114.739
1	1	C Epigraph	0.59591	200.809	0.73525	59.040
2	2	M Epigraph	0.57552	682.704	0.76864	230.593
2	2	C Epigraph	0.70760	558.608	0.84494	204.578
3	3	C Epigraph	0.73852	778.603	0.88154	338.417
2	6	C Tailored	0.73440	527.623	0.87996	191.538
3	6	C Tailored	0.76513	767.759	0.90373	290.985

(c) Evaluated against M-group

			Gag		p24	
n	*m*	Vaccine	Coverage	Extras	Coverage	Extras
1	1	M Epigraph	0.46416	289.843	0.59210	93.267
2	2	M Epigraph	0.62121	664.677	0.78726	229.504
3	3	M Epigraph	0.67648	931.225	0.83539	299.959
2	6	M Tailored	0.68690	621.867	0.84368	214.370
3	6	M Tailored	0.71526	909.538	0.87381	313.950

Table 6. Conserved regions selected for vaccine design. Numbering is relative to proteins in the reference strain Zaire 1976 EBOV virus, NC_002549. See Fig. 4A in the main text for more detail.

Protein	start	stop	length
Nucleoprotein	132	410	279
Matrix	71	193	123
Polymerase.1	540	854	314
Polymerase.3	952	1060	109
Total: 825 amino acids			

Table 7. Ebola coverage: A summary of coverage statistics for a subset of Ebola Epigraph sequence options we explored. Solution A is the best single Epigraph using the 5 Ebola species set (Epigraph a). B is based on fixing "a", and complementing it with an Epigraph that will give the best coverage of the 34 outbreak sequence set (Epigraphs a+b). C is the 2 Epigraph solution, simultaneously solved, for the 34 outbreak set (Epigraphs c1+c2). D fixes the two Epigraphs in B, and adds a third complementary sequence to improve coverage of the 34 outbreak sequences (Epigraphs a+b+d). E fixes epigraph A, and then simultaneously solves for 2 more complementary sequences that maximize coverage of the 34 outbreak sequences, for a total of 3 antigens (Epigraphs a+e1+e2). F is the simultaneously solved 3 Epigraph set that best covers the 34 outbreak sequences (Epigraphs f1+f2+f3). E is the strategy that we preferred, because it improves coverage of diverse sequences in rare species while maintaining excellent coverage of recurrent outbreak forms. Note that E will not perfectly match the optimal solution for the 34 outbreak sequences, solution F, but we preferred solution E as it provided better coverage of rarer species of *Ebolavirus*, with negligible loss of EBOV or SUDV coverage.

	Optimization:		exact	exact	$\lambda = 0.1$	$\lambda = 0.1$		
	Evaluation:		9/9	8/9	9/9	8/9		
Protein	vaccine	*m*					price	gain
Nucleoprotein	A	1	0.4953	0.6196	0.4858	0.6270	-0.0096	0.0074
Nucleoprotein	B	2	0.5571	0.6365	0.5545	0.6419	-0.0026	0.0053
Nucleoprotein	C	2	0.6919	0.7920	0.6815	0.7953	-0.0104	0.0033
Nucleoprotein	D	3	0.6830	0.7410	0.6765	0.7498	-0.0065	0.0088
Nucleoprotein	E	3	0.7490	0.8334	0.7369	0.8389	-0.0121	0.0054
Nucleoprotein	F	3	0.8206	0.9047	0.8106	0.9077	-0.0100	0.0030
Region1	A	1	0.8052	0.9461	0.7897	0.9535	-0.0155	0.0074
Region1	B	2	0.8619	0.9491	0.8781	0.9651	0.0162	0.0160
Region1	C	2	0.8850	0.9651	0.8781	0.9651	-0.0068	0.0000
Region1	D	3	0.9361	0.9808	0.9362	0.9875	0.0001	0.0067
Region1	E	3	0.9361	0.9808	0.9362	0.9875	0.0001	0.0067
Region1	F	3	0.9503	0.9851	0.9350	0.9889	-0.0153	0.0038
Region2	A	1	0.7635	0.9496	0.7461	0.9530	-0.0174	0.0035
Region2	B	2	0.8061	0.9345	0.7954	0.9350	-0.0107	0.0005
Region2	C	2	0.8430	0.9678	0.8430	0.9678	0.0000	0.0000
Region2	D	3	0.9223	0.9775	0.8721	0.9957	-0.0501	0.0182
Region2	E	3	0.9223	0.9775	0.8721	0.9957	-0.0501	0.0182
Region2	F	3	0.9384	0.9693	0.8721	0.9957	-0.0662	0.0263
Region3	A	1	0.7674	0.9114	0.7550	0.9231	-0.0124	0.0117
Region3	B	2	0.8357	0.9434	0.8328	0.9517	-0.0029	0.0083
Region3	C	2	0.8693	0.9505	0.8547	0.9593	-0.0147	0.0088
Region3	D	3	0.9280	0.9831	0.9202	0.9838	-0.0078	0.0007
Region3	E	3	0.9280	0.9831	0.9202	0.9838	-0.0078	0.0007
Region3	F	3	0.9449	0.9854	0.9228	0.9875	-0.0221	0.0021
Region4	A	1	0.7980	0.9762	0.7505	0.9822	-0.0475	0.0059
Region4	B	2	0.8235	0.9732	0.7991	0.9703	-0.0245	-0.0029
Region4	C	2	0.8416	0.9505	0.8235	0.9732	-0.0181	0.0227
Region4	D	3	0.9275	0.9819	0.8905	0.9881	-0.0370	0.0061
Region4	E	3	0.9275	0.9819	0.8905	0.9881	-0.0370	0.0061
Region4	F	3	0.9464	0.9840	0.8905	0.9881	-0.0559	0.0041
Spike	A	1	0.3883	0.5400	0.3659	0.5531	-0.0224	0.0132
Spike	B	2	0.4214	0.4969	0.3993	0.5357	-0.0221	0.0388
Spike	C	2	0.5644	0.6939	0.5506	0.7006	-0.0138	0.0068
Spike	D	3	0.5877	0.6715	0.5792	0.6830	-0.0085	0.0115
Spike	E	3	0.6504	0.7633	0.6281	0.7666	-0.0223	0.0033
Spike	F	3	0.7285	0.8376	0.7214	0.8397	-0.0071	0.0022

Table 8. Computation. Run times, in seconds, for the Epigraph algorithm on a modern laptop computer. The basic run ($m = 1$) creates a graph from the sequence data, removes cycles, and then uses the dynamical programming scheme in Eq. (2) and Eq. (3) to find the best path. Because the dynamical programming step is so fast, there is very little marginal cost in finding a second path, using the sequential approach, without ($m = 1+1$) or with ($m = 2$) iterative refinement. The last column shows the cost of computing new pairs of antigens using iterative refinement with $T = 100$ random initializations for the first path. (Note the Env E data sample does not permit a solution with rare epitopes excluded.)

Protein and Clade	Number of Sequences	Number of Distinct PTEs	$m = 1$ (basic)	$m = 1+1$ (sequential)	$m = 2$ (iterative)	$m = 2$ $T = 100$
Exclude rare epitopes: $n_o = 1$						
Gag E	673	8416	1.29	1.33	1.34	13.66
Gag B	1729	17273	3.04	3.15	3.20	25.75
Gag C	940	12823	2.18	2.17	2.21	20.93
Gag M	4596	45404	8.27	8.48	8.91	73.26
Nef E	246	2894	0.55	0.57	0.58	3.83
Nef B	1780	14522	1.81	1.86	1.92	17.37
Nef C	749	7523	1.01	1.04	1.09	8.85
Nef M	4040	30879	4.09	4.33	4.55	48.23
Pol E	348	7315	1.26	1.29	1.33	15.03
Pol B	1072	19732	3.26	3.37	3.40	38.20
Pol C	414	10882	1.75	1.84	1.86	19.77
Pol M	2780	42048	7.89	8.17	8.51	90.40
Env B	1433	58320	7.77	8.07	8.07	89.32
Env C	1124	45221	5.67	5.86	6.11	65.05
Env E	420	17825	–	–	–	–
Env M	4250	152343	25.79	26.92	29.09	381.47
Include all epitopes: $n_o = 0$						
Gag E	673	22107	3.98	3.62	3.69	43.11
Gag B	1729	48297	15.38	15.42	15.83	102.67
Gag C	940	35166	6.82	6.63	7.40	67.80
Gag M	4596	128940	52.01	56.44	50.23	366.43
Nef E	246	8248	1.53	1.59	1.61	13.09
Nef B	1780	44497	6.93	6.83	7.60	85.47
Nef C	749	21639	3.03	3.15	3.35	38.77
Nef M	4040	90850	16.95	16.03	17.01	223.88
Pol E	348	17749	2.48	2.70	2.90	38.44
Pol B	1072	45381	10.28	10.59	11.17	98.29
Pol C	414	26632	4.44	4.49	5.00	53.01
Pol M	2780	103471	38.19	38.18	40.33	395.15
Env B	1433	254412	123.29	137.06	210.90	997.47
Env C	1124	200964	64.72	82.08	72.15	618.25
Env E	420	71841	11.87	11.30	12.53	194.43
Env M	4250	666137	1063.41	1161.57	1260.84	4473.97

Table 9. Compare unaligned and aligned epigraphs. This table contains no new information, but combines results from Table 2 and Table 3 to display side-by-side the performance of unaligned and aligned epigraphs. In general the differences are small, with the largest difference, for the $m = 2$ Nef C case, just over 0.01. For Gag, Nef, and Env, we see that the unaligned performance is almost always better. For Pol, the aligned performance is often better though it bears remarking that for Pol, the differences between the two are particularly small, always less than 0.0005. Asterisks indicate larger values.

Protein and Clade	$m = 1$		$m = 1+1$		$m = 2$	
	Unaligned	Aligned	Unaligned	Aligned	Unaligned	Aligned
Gag E	0.687068	0.687068	0.770351*	0.769894	0.770971*	0.770523
Gag B	0.613491*	0.613473	0.727084*	0.726896	0.727091*	0.726903
Gag C	0.603367*	0.602685	0.711297*	0.711228	0.711813*	0.711259
Gag M	0.464157	0.465539*	0.620641*	0.619661	0.621405*	0.620100
Nef E	0.456775*	0.450319	0.596507*	0.586679	0.596507*	0.586741
Nef B	0.363338*	0.362313	0.480489*	0.477803	0.482001*	0.479306
Nef C	0.390911*	0.384473	0.527535*	0.517049	0.527750*	0.517263
Nef M	0.285584*	0.279744	0.396834*	0.387946	0.398410*	0.388959
Pol E	0.791086	0.791109*	0.866522*	0.866443	0.866639*	0.866493
Pol B	0.703722	0.703968*	0.798906	0.798931*	0.798906	0.798931*
Pol C	0.731726	0.731857*	0.817636	0.817685*	0.817636	0.817685*
Pol M	0.612558	0.612590*	0.734062	0.734103*	0.735260	0.735691*
Env B	0.379334*	0.379268	0.468086*	0.467930	0.468251*	0.467930
Env C	0.397204*	0.397142	0.490135*	0.489724	0.490454*	0.489988
Env E	0.493401*	0.493353	0.577352*	0.576248	0.577467*	0.576248
Env M	0.282244*	0.281127	0.379740*	0.377815	0.380971*	0.379208
